# Stereotactic Neuro-Navigation Phantom Designs: A Systematic Review

**DOI:** 10.3389/fnbot.2020.549603

**Published:** 2020-10-23

**Authors:** Marko Švaco, Ivan Stiperski, Domagoj Dlaka, Filip Šuligoj, Bojan Jerbić, Darko Chudy, Marina Raguž

**Affiliations:** ^1^Faculty of Mechanical Engineering and Naval Architecture, University of Zagreb, Zagreb, Croatia; ^2^Department of Neurosurgery, University Hospital Dubrava, Zagreb, Croatia; ^3^Croatian Institute for Brain Research, School of Medicine University of Zagreb, Zagreb, Croatia; ^4^Department of Surgery, School of Medicine University of Zagreb, Zagreb, Croatia; ^5^Department of Anatomy and Clinical Anatomy, School of Medicine University of Zagreb, Zagreb, Croatia

**Keywords:** robotics, head phantom, stereotactic neurosurgery, neuronavigation, target point error, entry point error, angular error

## Abstract

Diverse stereotactic neuro-navigation systems are used daily in neurosurgery and novel systems are continuously being developed. Prior to clinical implementation of new surgical tools, methods or instruments, *in vitro* experiments on phantoms should be conducted. A stereotactic neuro-navigation phantom denotes a rigid or deformable structure resembling the cranium with the intracranial area. The use of phantoms is essential for the testing of complete procedures and their workflows, as well as for the final validation of the application accuracy. The aim of this study is to provide a systematic review of stereotactic neuro-navigation phantom designs, to identify their most relevant features, and to identify methodologies for measuring the target point error, the entry point error, and the angular error (α). The literature on phantom designs used for evaluating the accuracy of stereotactic neuro-navigation systems, i.e., robotic navigation systems, stereotactic frames, frameless navigation systems, and aiming devices, was searched. Eligible articles among the articles written in English in the period 2000–2020 were identified through the electronic databases PubMed, IEEE, Web of Science, and Scopus. The majority of phantom designs presented in those articles provide a suitable methodology for measuring the target point error, while there is a lack of objective measurements of the entry point error and angular error. We identified the need for a universal phantom design, which would be compatible with most common imaging techniques (e.g., computed tomography and magnetic resonance imaging) and suitable for simultaneous measurement of the target point, entry point, and angular errors.

## Introduction

**Figure 1:**
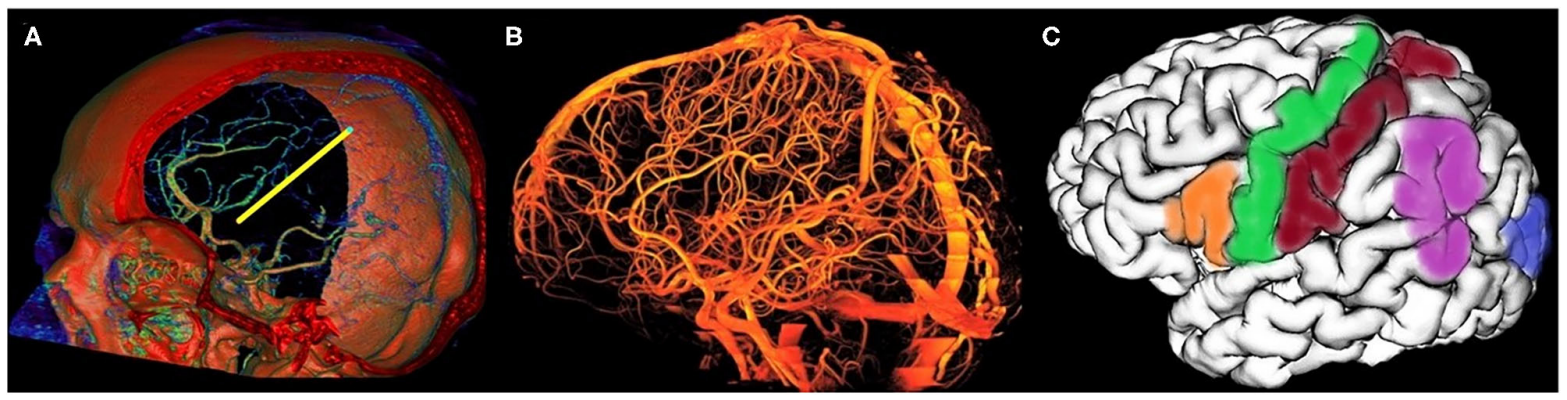
Avoiding all visible vessels is critical in stereotactic planning. **(A)** A 3D reconstruction of human cerebral arterial system with a planned trajectory depicted in yellow—the planned trajectory is shown from the stereotactic planning module RONNAplan (Jerbić et al., 2020); **(B)** a 3D reconstruction of a time of flight magnetic resonance angiography showing a complete vascular system of the human brain; **(C)** a 3D MRI reconstruction of left cerebral hemisphere with eloquent brain areas marked in colors (green—motor area, red—sensory area, blue—visual area, orange—Broca's area, and purple—Wernicke's area).

In the vast majority of keyhole stereotactic neurosurgical interventions, namely biopsies, deep brain stimulation (DBS), stereoelectroencephalography (SEEG), ventricular puncture, and catheter placement, straight cylindrical non-deformable instruments are introduced into the intracranial region of interest, aiming at the planned target. The primary objective of any keyhole neurosurgical procedure is to reach the planned target with minimal deviation, i.e., targeting error, while avoiding blood vessels (Figures 1A,B). Furthermore, it is necessary to avoid functional and eloquent brain areas, such as the sensory and motor cortex areas, eloquent temporal regions, and the primary visual cortex (Figure 1C). By avoiding visible vessels and critical areas within the brain while navigating through a narrow entry point (two to 30 millimeters in diameter) toward the target point ensures maximum safety and minimizes potential complications. The diameter of the instruments ranges from two (various probes and electrodes) to roughly 15 mm (larger tools such as endoscopes), while the target size can vary significantly, from a few millimeters to a couple of centimeters (e.g., large tumors).

Before being applied in clinical practice, all stereotactic robotic, frameless, and frame-based neuro-navigation devices require rigorous preclinical testing on phantom setups. A stereotactic neuro-navigation phantom denotes a rigid or deformable structure resembling the cranium with the intracranial area. The use of phantoms is essential for the testing of complete procedures and their workflows, as well as for the final validation of the application accuracy. A typical stereotactic neuro-navigation procedure consists of a preoperative, a preparation, and an operative phase. In the preoperative phase, the imaging localizers are attached to the patient in frame-based and fiducial-based procedures; in some systems, there is no need for localizers (markers) when markerless registration is used. In the next step, preoperative imaging is performed (computed tomography—CT or magnetic resonance imaging—MRI), followed by the planning of surgical trajectories, which are commonly composed of entry and target points. In the preparation phase, localization in the operating theater and registration with preoperative images are performed. The operative phase consists of instrument navigation and targeting within the intracranial space. The targeting accuracy, which ensures the technical success of the stereotactic procedure and the patient's safety, is of crucial importance for clinical application; it is assessed in a number of phantom and patient studies (Widmann and Bale, 2006; Lozano, 2009; Widmann et al., 2009; Cardinale et al., 2013; González-Martínez et al., 2016; Švaco et al., 2017a,b; Dlaka et al., 2018). Phantom features, i.e., the distribution of localization and targeting elements, should correspond as close as possible to those used in actual operation procedures performed on patients. Additionally, the position of these elements should be determined by standard imaging techniques and localization devices used for preoperative imaging or by localization devices in the operating room.

**Figure 2:**
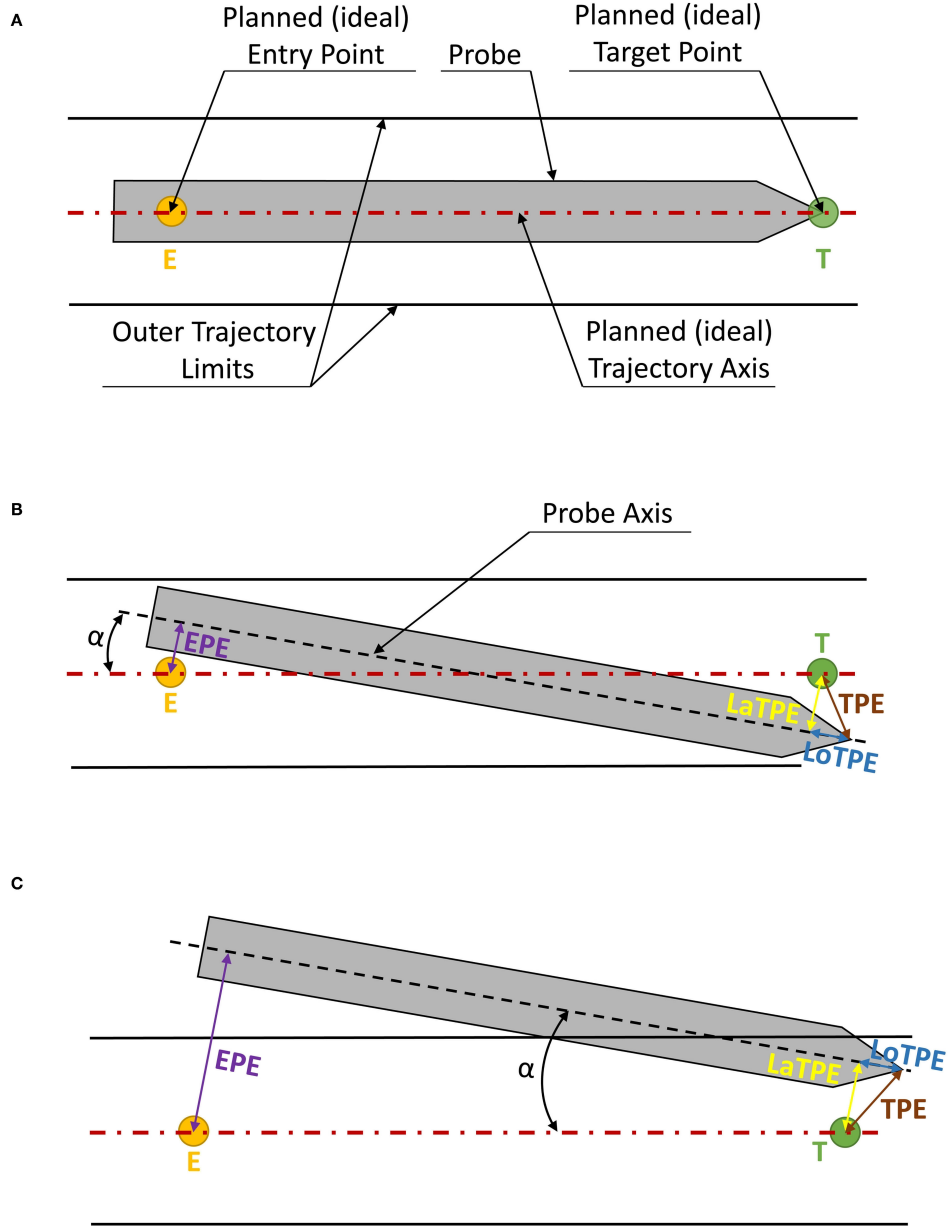
Types of errors measured at the target and entry points. The operative instrument is indicated in gray, the red dotted line indicates the ideal trajectory going through the planned entry point (E) and the target point (T), while the two parallel black lines indicate the “no-go” zone, i.e., the cylinder in which the targeting error of the operative instrument is permissible. The longitudinal target point error (LoTPE) is indicated as a solid blue line; the lateral target point error (LaTPE) as a yellow line; the total TPE, derived from the Pythagoras theorem from LoTPE and LaTPE, is indicated as a brown line; the entry point error (EPE) is indicated as a magenta solid line. **(A)** An ideal probe position with no errors; **(B)** A scenario with LoTPE, LaTPE, α, and EPE; **(C)** TPE and α are of the same magnitude, but the EPE is much greater due to the alignment of the lateral errors.

Assessment and standardization of errors is essential in the development, testing, and clinical application of computer-assisted neurosurgery systems. The target registration error (TRE) and the target point error (TPE) in particular have been recognized as the most important error measurements in computer-assisted surgery (Widmann et al., 2009). TRE is defined as the error between corresponding target points in the image data and the patient after registration. TPE is defined as the mismatch between the position of a puncture device guided during the actual surgical procedure and the preoperatively planned position of the surgical target. TPE refers to the application error during computer-assisted targeting, such as in brain biopsy, DBS, and SEEG. It is influenced by all errors committed during the surgical procedure, such as localization inaccuracies, hand tremor and movement errors of the operator during navigation, brain shift, and inaccuracies of the tool calibration. TPE can be measured through its two components (Figure 2): (a) the lateral (radial) error (depicted as LaTPE), which is the perpendicular distance from the target to the surgical tool axis, and (b) the longitudinal (depth) error (depicted as LoTPE), which is the distance on the tool axis between the surgical tool tip and the perpendicular axis at the target. These two errors form the total TPE which is defined as the Euclidean distance, i.e., the difference between the planned target point and the actual tool tip position calculated from Pythagoras' theorem. When the TPE is mentioned further in the text, it refers to the total TPE calculated from the LaTPE and LoTPE. Other important errors in neurosurgical targeting are the entry point error (EPE) and the angular error (α) (Widmann et al., 2009). The EPE is defined as the perpendicular distance between the planned entry point and the probe (instrument) axis (Figure 2). The EPE is a significant factor in accuracy measurement in *in vivo* studies, while it is still mostly neglected in phantom studies (Cardinale et al., 2013; González-Martínez et al., 2016; Dlaka et al., 2018). For measuring the EPE in a clinical scenario, image fusion of the postoperative CT scan and the preoperative CT or MRI scans can be made. The EPE is then measured as previously mentioned and as shown in Figure 2. The angular error, α, is defined as the angular deviation of the centerline of the actual trajectory from the centerline of the ideal, i.e., planned trajectory. When the values of both the lateral and longitudinal TPEs and of angular error, α, are identical, the EPE could have a significant error range (see Figures 2B,C). Thus, we believe that all targeting errors (TPE, EPE, and α) are important in neurosurgical targeting and should be assessed in preclinical and laboratory phantom studies. In addition, there are still no reported standards for the targeting error assessment and no universal phantom design suitable for objective measurement of TPE, EPE, and α.

The aim of this study is to provide a systematic review of stereotactic neuro-navigation phantom designs and to identify their most relevant design features, such as their size and shape, material, filling, entry and target point design, compatibility with standard stereotactic frames and head holders, compatibility with standard localization and registration methods, and compatibility with devices for measuring the targeting error. All these features are presented in detail in the stereotactic phantom survey. Furthermore, we have made a detailed review of methodologies used for measuring the three targeting errors (TPE, EPE, and α) on phantoms.

## Methods

**Figure 3:**
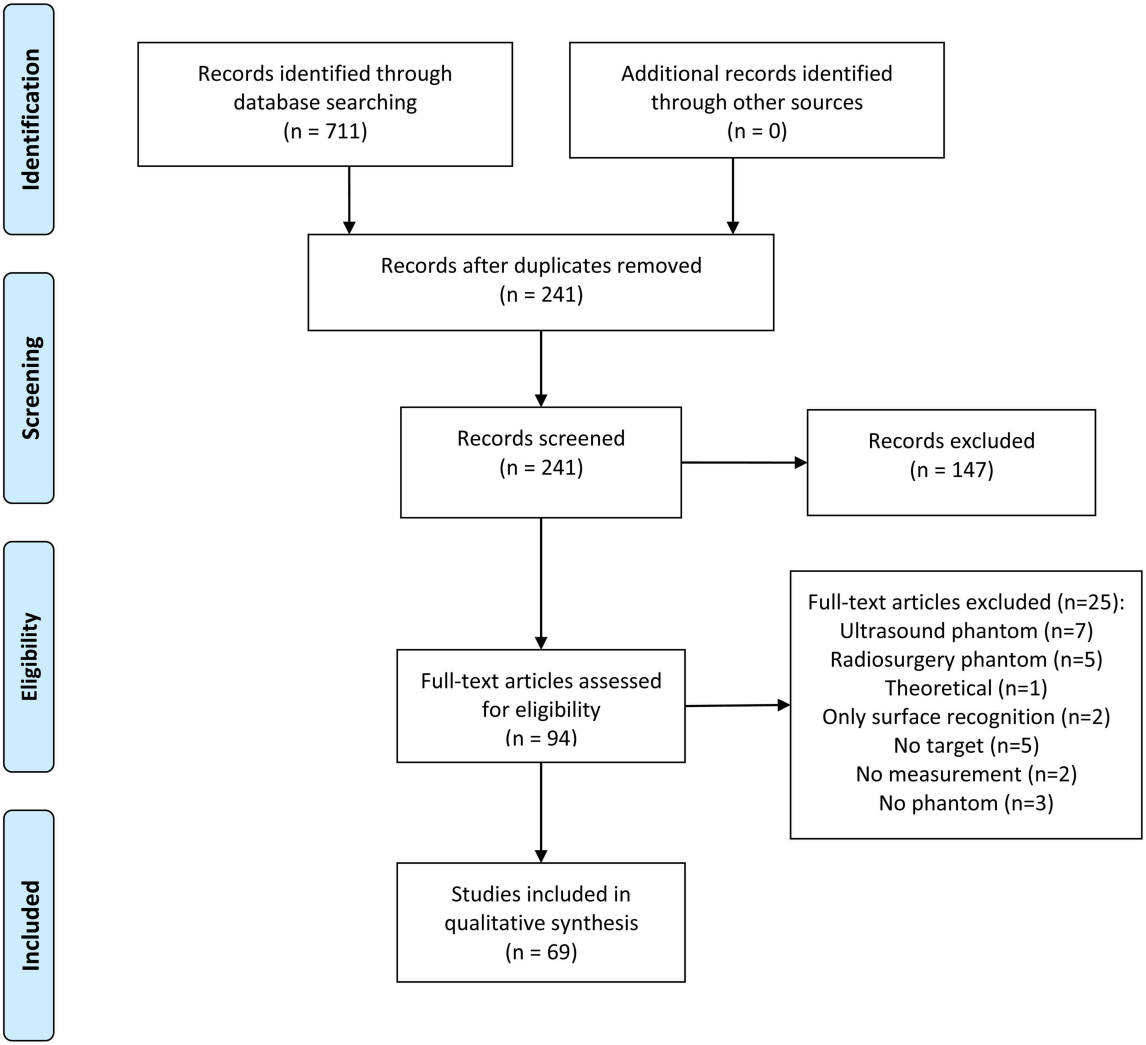
PRISMA flowchart for the selection of articles.

A systematic review of stereotactic neuro-navigation phantom designs used for evaluating the accuracy of neuro-navigation systems (robotic neuro-navigation systems, stereotactic frames, frameless navigation systems, aiming devices) was done. M.Š. and I.S. independently searched the PubMed, IEEE, Web of Science, and Scopus databases for publications over a 20-years period between January 1, 2000 and March 25, 2020. Search terms were generated using the PICO tool (Problem, Intervention, Comparison, and Outcome) and the free text searching was done using the Boolean free-text search: [(Phantom OR Head Phantom) AND (Robot^*^ OR stereotaxy OR imaging system OR navigation system OR frame^*^) AND (Neurosurg^*^ OR Neuronavigation) AND (Accuracy OR Target point OR Measurement^*^ OR Error^*^)]. The last search was carried out on March 25, 2020. The reference lists of selected studies were also investigated in order to identify additional eligible publications. Duplicates were then removed, and an English language restriction was applied. Titles and abstracts were screened to identify publications that met the following criteria: (1) Phantoms representing the human head, (2) Phantoms that are clearly described, (3) Phantoms having at least one target point, (4) Phantoms used to test a neuro-navigation procedure, and (5) Accuracy data being provided. Full versions of publications were then obtained and assessed for further selection. Any discrepancies were resolved by consensus and in discussion with the senior authors. Seventy papers were finally included in the quantitative synthesis. Figure 3 shows the article selection flowchart made according to the PRISMA guidelines (Moher et al., 2009).

**Figure 4:**
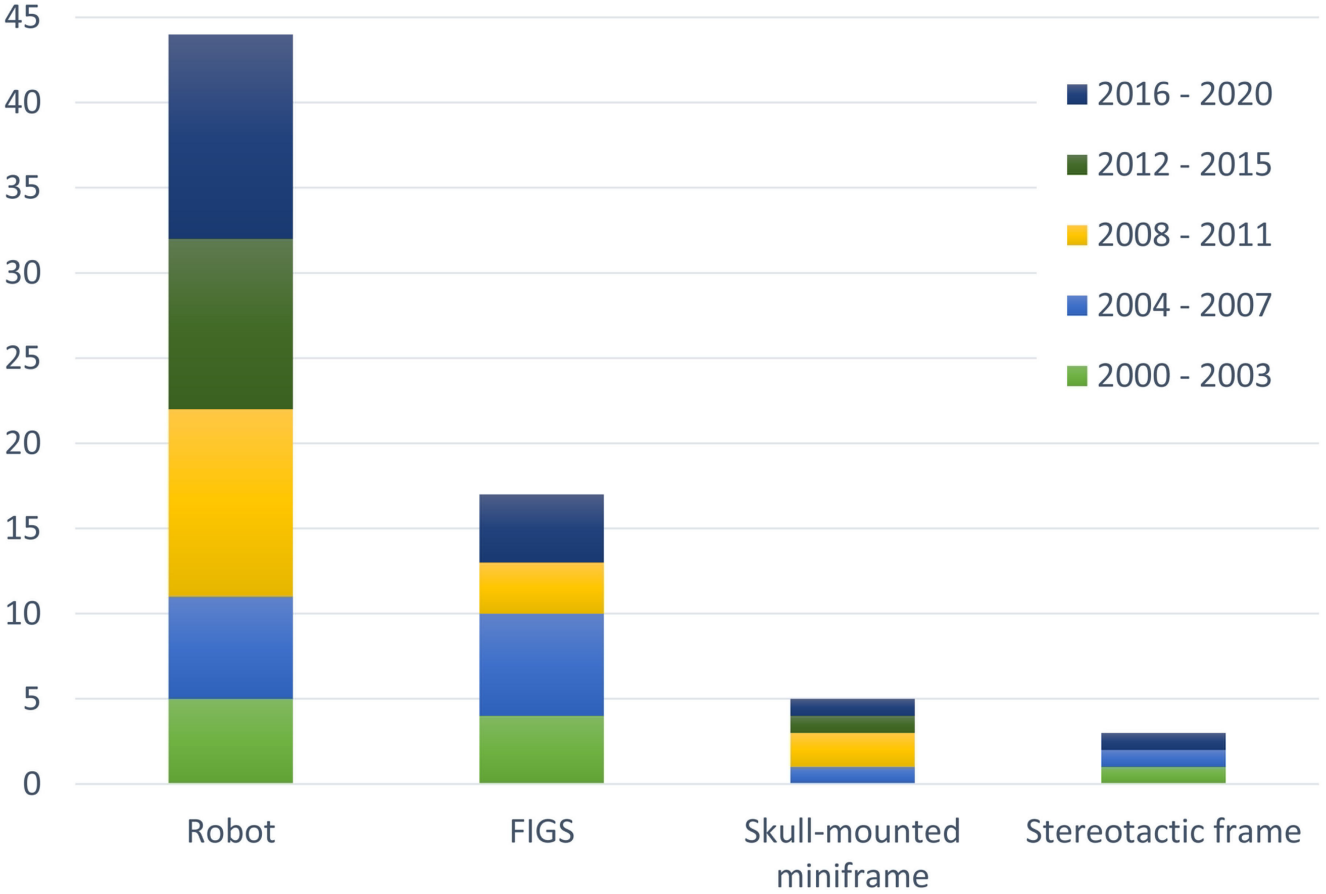
The number of phantom designs developed for four major types of neuro-navigation systems: robot, frameless image guided system (FIGS), skull-mounted miniframe, and stereotactic frame. In total, 63% of all phantom designs are developed for robotic neuro-navigation systems. Since 2010 the percentage is even higher, with 78% of all phantom designs developed for robotic systems. Although phantom designs are mostly developed to test the targeting accuracy of robotic neuro-navigation devices, the majority of phantoms can be used to test the three remaining categories of neuro-navigation systems.

We have included references from the period of 20 years, i.e., from 2000 to 2020, presented in the text and accompanied by Figures 4, 6, in particular. Additionally, due to a large amount of published data, only the phantom designs from the 2010–2020 decade were included in the Supplementary Table 1 in order to present the latest and detailed information regarding phantom design features.

## Stereotactic Phantom Survey

Through the literature review, we have identified relevant features and limitations of current phantom designs used in the field of robotic, frameless, and frame-based stereotactic neuro-navigation. These relevant features describe the key physical (anatomical) and functional properties of stereotactic neuro-navigation phantom designs. They define how well a phantom describes the area of interest during surgery, i.e., the cranium with the intracranial area, and how accurately the phantom can simulate the actual surgical procedure.

We have identified seven relevant features of phantom designs: (1) size and shape, (2) material, (3) filling, (4) entry and target design, (5) compatibility with standard stereotactic frames and head holders, (6) compatibility with standard registration and localization methods, and (7) compatibility with devices for measuring the targeting error. Furthermore, we have summarized the number and the type of targets in **Figure 6**. We have not systematically reported on the size of targets because it is closely related to the type of targets used and it cannot be clearly compared. Characteristics such as position and mechanical properties of targets are listed in the Supplementary Table 1. Given a large number of identified papers within the selected period, a detailed review of phantom designs is given in the Supplementary Table 1 only for a 10 year-period (2010–2020). Furthermore, for each relevant feature, we have chosen particular phantom designs which we found the most representative. In each of the following seven subsections, these phantom designs are presented in more detail.

### Size and Shape

**Figure 5:**
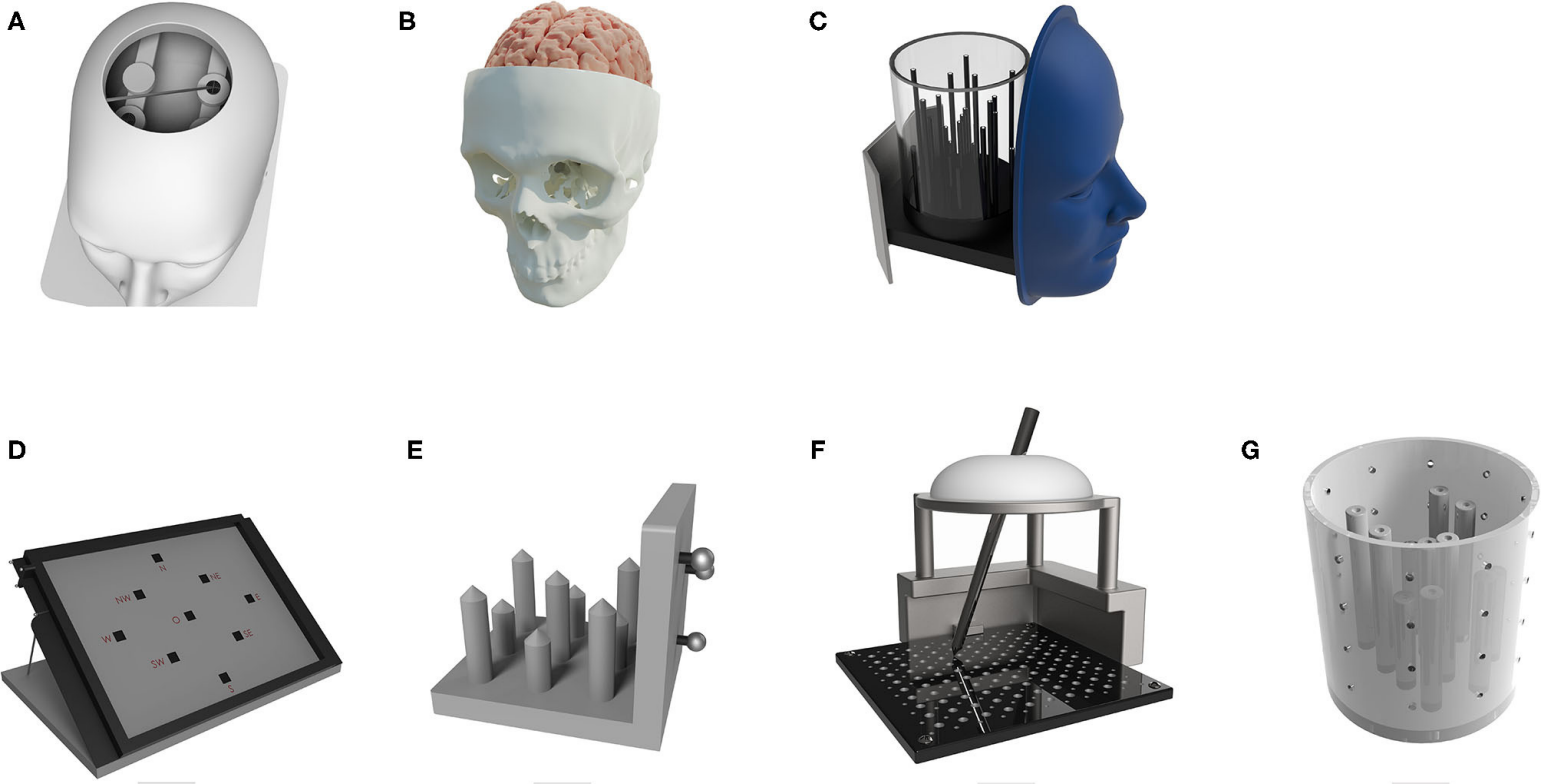
Various phantom designs—anthropomorphic phantom designs **(A–C)** and non-anthropomorphic phantoms **(D–G)**: **(A)** a plastic skull phantom fitted with two metal discs as target points and a star-shaped base plate used for Renaissance Robot measurements (Joskowicz et al., 2011); **(B)** a plastic skull replica phantom with a polyvinyl alcohol brain structure and two gadolinium markers as target points (Comparetti et al., 2012); **(C)** a plastic phantom with a circular base fitted with acrylic tubes used as target points; the phantom has a removable container for water and a face replica for surface registration technique; the phantom was tested on the ROSA Brain system (Lefranc et al., 2014); **(D)** a phantom concept made of a paper sheet with printed target points used for robotic neuro-navigation based on a Kuka robot (Niccolini et al., 2016); **(E)** a non-anthropomorphic phantom with plastic target point tips used for a robotic system based on DLR/KUKA Lightweight robot III (Tovar-Arriaga et al., 2011); **(F)** a phantom made of a vinyl sheet with accurately milled reference points which served as target points for testing a skull-mounted micro stereotactic frame (Rau et al., 2017); and **(G)** an organic glass cylindrical phantom with rods placed as target points used for a surgical robot for stereotactic biopsy testing; the volume of the phantom was approximately the same as the human head volume (Meng et al., 2014).

The human brain volume is approximately between 1,260 and 1,443 cm^3^ (Lüders et al., 2002). The phantom volume should be similar to the actual human brain volume in order to make studies conducted on phantoms comparable to cadaveric and patient studies. Phantoms are classified as *anthropomorphic* and *non-anthropomorphic* phantoms. An anthropomorphic phantom consists of a standard human skull replica or head mold with custom-made target points (Table 1), while a non-anthropomorphic phantom is usually made of cylindrical or prismatic containers, approximating the targeted human brain volume (Table 1). An example of a non-anthropomorphic phantom composed of a vinyl sheet was reported in the study by Rau et al. (2017). A grid of accurately milled reference marks with a 10 mm intermediate distance between the reference marks was used to measure targeting errors of a skull-mounted micro-stereotactic frame (Figure 5F). Measurements were taken using a reflected-light microscope (Leica APO Z6, Leica Microsystems GmbH, Wetzlar, Germany). The main disadvantage of that phantom design is the inaccurate representation of the skull and the targeted anatomy; as a result, it is impossible to measure the target longitudinal error and EPE. Furthermore, a two dimensional targeting plate does not accurately represent the actual intracranial volume. Although non-anthropomorphic phantoms are convenient and economical to produce, simulation of the human anatomy is inaccurate, which represents a significant drawback, especially in the registration process (Moriarty et al., 2000; Benardete et al., 2001; Poggi et al., 2003; Krempien et al., 2004).

**Table 1:** References describing each of the seven relevant phantom features.

**Phantom design features**	**References**
Size and shape	**Anthropomorphic phantoms:** Landi et al., 2001; Liu et al., 2001; Li et al., 2002; Amin and Lunsford, 2004; Henderson, 2004; Henderson et al., 2004; Labadie et al., 2005; Pappas et al., 2005; Quiñones-Hinojosa et al., 2006; Rosenow and Sootsman, 2006; Shamir et al., 2006; Varma and Eldridge, 2006; Eggers and Muhling, 2007; Eljamel, 2007; Rachinger et al., 2007; Widmann et al., 2008; Xia et al., 2008; Balachandran et al., 2009; Arata et al., 2011; Brodie and Eljamel, 2011; Joskowicz et al., 2011; Comparetti et al., 2012; Heinig et al., 2012; Larson et al., 2012; Lefranc et al., 2014; Kajita et al., 2015; Ballesteros-Zebadúa et al., 2016; Lin et al., 2016; Niccolini et al., 2016; Cardinale et al., 2017; Cutolo et al., 2017; Zeng et al., 2017 **Non-anthropomorphic phantoms:** Moriarty et al., 2000; Reinges et al., 2000; Steinmeier et al., 2000; Benardete et al., 2001; Willems et al., 2001; Morgan et al., 2003; Poggi et al., 2003; Krempien et al., 2004; Bale et al., 2006; Liu et al., 2006, 2007; Chan et al., 2009; Labadie et al., 2009; Ringel et al., 2009; Stoffner et al., 2009; Baron et al., 2010; Schouten et al., 2010; Kratchman et al., 2011; Tovar-Arriaga et al., 2011; Gerber et al., 2013; Schulz et al., 2013; Meng et al., 2014; Šuligoj et al., 2015, 2017; Niccolini et al., 2016; Švaco et al., 2016; Cifuentes et al., 2017; Rau et al., 2017
Material	**CT:** Steinmeier et al., 2000; Liu et al., 2001, 2006, 2007; Morgan et al., 2003; Amin and Lunsford, 2004; Krempien et al., 2004; Labadie et al., 2005, 2009; Pappas et al., 2005; Varma and Eldridge, 2006; Eggers and Muhling, 2007; Rachinger et al., 2007; Widmann et al., 2008; Xia et al., 2008; Balachandran et al., 2009; Chan et al., 2009; Ringel et al., 2009; Stoffner et al., 2009; Baron et al., 2010; Giese et al., 2010; Brodie and Eljamel, 2011; Joskowicz et al., 2011; Kratchman et al., 2011 **CBCT, fpCT**: Gerber et al., 2013; Schulz et al., 2013; Lefranc et al., 2014; Cardinale et al., 2017 **MRI:** Moriarty et al., 2000; Reinges et al., 2000; Steinmeier et al., 2000; Benardete et al., 2001; Henderson et al., 2004; Rosenow and Sootsman, 2006; Widmann et al., 2008; Giese et al., 2010; Schouten et al., 2010; Comparetti et al., 2012; Kronreif et al., 2012 **Plexiglas:** Reinges et al., 2000; Steinmeier et al., 2000; Willems et al., 2001; Morgan et al., 2003; Poggi et al., 2003; Labadie et al., 2005; Widmann et al., 2008; Chan et al., 2009; Stoffner et al., 2009; Baron et al., 2010; Kratchman et al., 2011; Kronreif et al., 2012; Gerber et al., 2013; Švaco et al., 2016; Cifuentes et al., 2017 **Other types of plastics:** Quiñones-Hinojosa et al., 2006; Eggers and Muhling, 2007; Rachinger et al., 2007; Xia et al., 2008; Balachandran et al., 2009; Arata et al., 2011; Brodie and Eljamel, 2011; Joskowicz et al., 2011; Tovar-Arriaga et al., 2011; Comparetti et al., 2012; Larson et al., 2012; Lefranc et al., 2014; Meng et al., 2014; Kajita et al., 2015; Šuligoj et al., 2015; Ballesteros-Zebadúa et al., 2016; Lin et al., 2016; Niccolini et al., 2016; Cardinale et al., 2017; Cutolo et al., 2017; Zeng et al., 2017
Filling	**Air:** Carter et al., 2000; Moriarty et al., 2000; Reinges et al., 2000; Steinmeier et al., 2000; Benardete et al., 2001; Mutic et al., 2001; Yu et al., 2001a; Henderson, 2004; Henderson et al., 2004; Krempien et al., 2004; Lavely et al., 2004; Wang et al., 2004; Bale et al., 2006; Liu et al., 2006, 2007; Rosenow and Sootsman, 2006; Varma and Eldridge, 2006; Eggers and Muhling, 2007; Rachinger et al., 2007; Isambert et al., 2008; Xia et al., 2008; Chan et al., 2009; Labadie et al., 2009; Ringel et al., 2009; Stoffner et al., 2009; **Agent enchanted water:** Widmann et al., 2009; Baron et al., 2010; Schouten et al., 2010; Arata et al., 2011; Brodie and Eljamel, 2011; Kratchman et al., 2011; Kronreif et al., 2012; Larson et al., 2012; Squires et al., 2014; **Agar gel:** Lefranc et al., 2014; Nakazawa et al., 2014; Kajita et al., 2015; Li et al., 2015; Cardinale et al., 2017
Entry and target designs	**Quantitative methods**: Moriarty et al., 2000; Steinmeier et al., 2000; Benardete et al., 2001; Landi et al., 2001; Liu et al., 2001, 2006, 2007; Willems et al., 2001; Morgan et al., 2003; Poggi et al., 2003; Amin and Lunsford, 2004; Henderson, 2004; Henderson et al., 2004; Krempien et al., 2004; Labadie et al., 2005, 2009; Pappas et al., 2005; Bale et al., 2006; Quiñones-Hinojosa et al., 2006; Rosenow and Sootsman, 2006; Shamir et al., 2006; Varma and Eldridge, 2006; Eggers and Muhling, 2007; Eljamel, 2007; Rachinger et al., 2007; Widmann et al., 2008, 2009; Balachandran et al., 2009; Chan et al., 2009; Ringel et al., 2009; Baron et al., 2010; Giese et al., 2010; Schouten et al., 2010; Arata et al., 2011; Brodie and Eljamel, 2011; Joskowicz et al., 2011; Kratchman et al., 2011; Tovar-Arriaga et al., 2011; Comparetti et al., 2012; Heinig et al., 2012; Kronreif et al., 2012; Larson et al., 2012; Gerber et al., 2013; Schulz et al., 2013; Lefranc et al., 2014; Meng et al., 2014; Kajita et al., 2015; Li et al., 2015; Ballesteros-Zebadúa et al., 2016; Lin et al., 2016; Niccolini et al., 2016; Švaco et al., 2016; Cardinale et al., 2017; Zeng et al., 2017; **Qualitatitve methods**: Cifuentes et al., 2017; Cutolo et al., 2017; Rau et al., 2017 **Anatomical targets:** Liu et al., 2001; Arata et al., 2011; Niccolini et al., 2016 **Non-anatomical targets:** Moriarty et al., 2000; Steinmeier et al., 2000; Benardete et al., 2001; Landi et al., 2001; Willems et al., 2001; Morgan et al., 2003; Poggi et al., 2003; Henderson, 2004; Henderson et al., 2004; Krempien et al., 2004; Pappas et al., 2005; Bale et al., 2006; Liu et al., 2006, 2007; Quiñones-Hinojosa et al., 2006; Rosenow and Sootsman, 2006; Varma and Eldridge, 2006; Eljamel, 2007; Widmann et al., 2008; Chan et al., 2009; Ringel et al., 2009; Schouten et al., 2010; Brodie and Eljamel, 2011; Kratchman et al., 2011; Tovar-Arriaga et al., 2011; Heinig et al., 2012; Kronreif et al., 2012; Schulz et al., 2013; Lefranc et al., 2014; Meng et al., 2014; Kajita et al., 2015; Li et al., 2015; Ballesteros-Zebadúa et al., 2016; Cardinale et al., 2017; Cifuentes et al., 2017; Cutolo et al., 2017; Zeng et al., 2017
Compatibility with standard stereotactic frames and head holders	**Leksell frame:** Carter et al., 2000; Landi et al., 2001; Yu et al., 2001a; Li et al., 2002; Henderson, 2004; Henderson et al., 2004; **Cosman-Robert-Wells frame:** Quiñones-Hinojosa et al., 2006; Eljamel, 2007; Heinig et al., 2011, 2012; **Zamorano-Dujovny frame:** Lefranc et al., 2014; Nakazawa et al., 2014; Kajita et al., 2015
Compatibility with standard localization and registration methods	Fitzpatrick, 2010; Markelj et al., 2012; Lefranc et al., 2014; Smith et al., 2014; Šuligoj et al., 2018a,b
Compatibility with devices for targeting error measurements	**Vernier calipers:** Reinges et al., 2000; Steinmeier et al., 2000; Landi et al., 2001; Liu et al., 2001; Willems et al., 2001; Li et al., 2002; Morgan et al., 2003; Poggi et al., 2003; Henderson et al., 2004; **Metal rulers:** Amin and Lunsford, 2004; Henderson, 2004; Labadie et al., 2005; Quiñones-Hinojosa et al., 2006; Rosenow and Sootsman, 2006; Shamir et al., 2006; Varma and Eldridge, 2006; **Depth gauges:** Bale et al., 2006; Liu et al., 2006; Eggers and Muhling, 2007; Eljamel, 2007; Rachinger et al., 2007; Rawlings and Crawford, 2008; Xia et al., 2008; Chan et al., 2009; Labadie et al., 2009; Ringel et al., 2009; Giese et al., 2010; Joskowicz et al., 2011; **CMM:** Brodie and Eljamel, 2011; Heinig et al., 2012; Larson et al., 2012; **Stereotactic frames:** Kronreif et al., 2012; Widmann et al., 2012; Gerber et al., 2013; Lefranc et al., 2014; Li et al., 2015; Šuligoj et al., 2015; **FIGS:** Kajita et al., 2015; Ballesteros-Zebadúa et al., 2016; Niccolini et al., 2016; **CT:** Ballesteros-Zebadúa et al., 2016; Švaco et al., 2016; **MRI:** Lin et al., 2016; Cutolo et al., 2017; Rau et al., 2017; Zeng et al., 2017

An anthropomorphic head phantom made of tissue and bone radio-transparent polymers in order to simulate an intracranial space was developed by Ballesteros-Zebadúa et al. (2016). The phantom size is roughly the same as the average human head size. Inside the phantom, an acrylic plate consisting of different acrylic shapes is placed. These shapes simulate internal surgical targets for image-guided surgery. Six rod and pyramid-shaped targets were placed on different locations inside the phantom cranial cavity. On the phantom cranial surface, six self-adhesive CT fiducial markers were attached for phantom registration purposes. The phantom was used to measure the accuracy of the Vector vision (BrainLab, Germany) frameless image-guided system (FIGS). The error measurements were taken using an optical system consisting of a calibrated pointer and a camera tracking system. This phantom can be used to validate surgical procedures such as brain biopsies because it has a proper soft tissue representation and a coarse estimation of a human head volume. It lacks the ability to measure the EPE, while different target shapes (rods and pyramids) introduce a small localization error in the planning phase of the procedure. Rods and pyramids have a defined center at their vertex (for pyramids) or at a center of a small circular surface (for rods). Given the geometrical resolution of CT scanners or other imaging systems, a vertex point cannot be clearly seen on the scan because of its infinite small size. A small circular plane in rod type targets directly borders with air and therefore the transition from the two materials is immediate. This introduces an error for the actual location of the surface in a scan given the geometrical resolution of the imaging system. Therefore, the localization of rod and pyramid target points is not fully accurate. The highest localization accuracy can be obtained by localizing spherical targets. Spherical targets have a predefined volume where the center of mass (center of all voxels belonging to a target) is actually in the center of the target sphere, so the error is homogeneous. Furthermore, spheres are identical in their cross section through their center when either orthogonal planes (axial, sagittal, and coronal) or multiplanar planes are used, because their cross section is always an ideal circle.

### Material

In every standard stereotactic procedure, imaging systems are used to obtain volumetric data of the patient for the preoperative planning phase. Thus, the materials used for making a phantom should have properties that comply with the characteristics of imaging systems. Widely used imaging systems are CT, CT-based imaging such as cone beam CT (CBCT) or flat-panel CT (fpCT), and MRI systems (Table 1). The phantoms should be compatible with these imaging techniques and should be able to go through a typical stereotactic neuro-navigation procedure, as described in the introduction. Due to specific demands of the imaging technology, choosing the adequate phantom material is of highest importance. Since the widely used imaging techniques have different working principles, the phantom material has to meet different requirements: either to partially absorb X-rays and partially allow X-ray passage avoiding potential artifacts on CT scans, or to contain increased amounts of water molecules for magnetic resonance imaging. In MRI, hydrogen atoms, naturally abundant in humans, in water or in fat, absorb radio frequency energy when placed in an external magnetic field. This results in an evolving spin polarization and induces a radio frequency signal detected in the MRI machine. Since the phantom structure must be rigid not to deform during handling and testing, and the materials should be non-toxic, Plexiglas and other types of plastics (Table 1) are widely used in CT imaging.

An example of a plastic phantom is described in Joskowicz et al. (2011). The authors of the study developed a plastic skull replica phantom placed on a star-shaped mounting base and fitted with two metal discs serving as target points (Figure 5A). Target discs are placed in positions to simulate deep brain targets. The top of the skull was cut to make target discs accessible, which resulted in the inability to measure the EPE. The centers of metal discs were marked, and concentric circles of 0.5 mm in diameter were drawn to allow the visual reading of target errors. The error is observed manually by a human operator through visual identification. In such measurements, human factors are present, and they can introduce parallax error if an improper angle of sight is used. The phantom was used to measure the accuracy of the Renaissance robotic system (*Mazor Robotics, Israel*). That phantom is suitable for measuring targeting errors for brain biopsies and deep brain stimulation (DBS) procedures. It has an opening around Kocher's point, which enables the insertion of biopsy needles and DBS electrodes into the target points. The metallic discs are a drawback since metals make artifacts in the imaging process during the localization, thus introducing additional measurement errors.

### Filling

The phantom filling represents the material that is found inside the phantom enclosure and defines the internal properties of the phantom, frequently simulating the brain tissue. The most commonly used filling is air (Table 1), which is sufficient for imaging systems such as CT and CT-based techniques. Other commonly used imaging systems, like MRI, need water to ensure imaging visibility. Thus, a water-based filling of the phantom is essential to produce an adequate MRI scan. In phantom designs, various agents are added to water to improve imaging visibility. In most cases, copper sulfate solutions are used (CuSO_4_∙ 5H_2_O) (Carter et al., 2000; Benardete et al., 2001; Yu et al., 2001a,b), along with some other widely used agents, such as gadolinium water solution (Steinmeier et al., 2000; Lefranc et al., 2014), copper sulfate and ^18^F-FDG (Isambert et al., 2008), solution of mixed gadolinium and iodinated contrast medium (Gd + I, 0.1 mmol/kg) (Nakazawa et al., 2014), 0.16 M CuSO_4_ solution, 30 mCi (1100MBq) ^99m^Tc solution, 6 mCi (220 MBq) ^18^F-2-fluoro-2 deoxyglucose (FDG) solution, trace amounts of FDG (Lavely et al., 2004), CuNO_3_ and ^18^F-fluorodeoxyglucose (^18^F-FDG) (Mutic et al., 2001), and water solution prepared according to the method recommended by the American Association of Physicists in Medicine (Wang et al., 2004). For a better brain tissue simulation, gelatin-based agar gel is used in some cases because its texture resembles the human brain tissue; this enables the insertion of electrodes and other surgical tools in the phantom (Moriarty et al., 2000; Stoffner et al., 2009; Widmann et al., 2009; Schouten et al., 2010; Larson et al., 2012; Squires et al., 2014; Li et al., 2015).

An example of a phantom filled with a gelatin-based agar gel representing the brain tissue is given by Schouten et al. (2010). In that phantom, target spheres were placed in the agar gel at a depth of three centimeters. A novel robotic needle guide manipulator was tested, and measurements were made after the needle placement on a postoperative MRI scan. This type of phantom filling enables a realistic postoperative procedure. For example, in DBS surgeries, after the DBS electrode is inserted into the sub-thalamic nucleus, a postoperative scan is performed in order to evaluate the correct positioning of the electrode. The agar gel filling is a good brain tissue representation due to its composition and material consistency, which enables the electrode to be inserted and postoperatively scanned. The target spheres were placed across the phantom volume. Although the postoperative TPE measurement introduces additional registration errors (MRI-to-MRI registration), it enables a fully realistic neurosurgical workflow replication.

### Entry and Target Design

One of the key steps in neurosurgical planning is the definition of the operative trajectory, which distinguishes two main points, an entry, and a target point. The targeting accuracy can be measured using quantitative or qualitative methods (Table 1). Quantitative measurements obtained from the phantom target points are mostly used for an *in vitro* accuracy evaluation of the whole system. Niccolini et al. (2016) developed a phantom (Figure 5D) made of a piece of paper with nine printed target points for measuring the quantitative targeting accuracy of a robotic system (*Kuka GmbH, Germany*). The paper with printed targets could be reoriented in order to obtain multiple phantom poses. The robot was navigated to the printed target points on the paper, then, it slightly perforated the paper with its operating tool, making small indents on the paper. The deviation of the indents from the printed targets was measured using a microscope (KH7700, Hirox Co., Tokyo, Japan), which enabled only lateral error measurements, and not the longitudinal TPE and EPE measurements (Niccolini et al., 2016).

A qualitative measurement does not include quantification in the form of an actual error measured in millimeters but mostly just as a categorization in the form of better/worse than a predefined error threshold. One often used approach in qualitative EPE measurement is in form of a burr hole on the phantom outer shell i.e., the skull surface (Table 1). In certain phantom experiments, the EPE is qualitatively determined by the following procedure: the burr hole has a known radius (the opening on the skull) and if the instrument is successfully inserted into the intracranial area, it is concluded that the lateral error is smaller than the difference between the burr hole radius and the tool radius. In order to properly evaluate the quantitative error using a burr hole it is necessary to make an orthogonal entry to the skull surface. If the instrument cannot be successfully inserted into the intracranial area because of collision with the phantom outer shell, the lateral error is greater than the difference between the burr hole radius and the tool radius.

A suitable target design for CT and MRI was used by Comparetti et al. (2012). The authors of the study developed an anthropomorphic plastic skull replica phantom depicted in Figure 5B. Inside the phantom, polyvinyl alcohol mimicked the brain structures, while at the base of the skull, two gadolinium markers served as target points. Due to its paramagnetic properties, gadolinium is widely used in neuroimaging; by changing the properties of tissue, gadolinium accumulates, causing enhancement of MRI. In addition, materials such as polyunsaturated oils are also widely used because of their chemical characteristics. The phantom was used for validation of the Robocast system (which consisted of a PathFinder robot—*PathFinder, Prosurgics Ltd., UK*, a Mazor SpineAssist robotic system -*SpineAssist, Mazor, Israel*, and a linear actuator—*Omega, Force Dimension, Switzerland*). The main advantages of this phantom are the anthropomorphic shape, the brain mimicking material such as water and agar gel, and the gadolinium target points, which enables the use of imaging methods for position error measurement. In order to obtain measurements, a coordinate measuring machine (CMM) and an optical measurement system (Optotrak Certus, Northern Digital Inc., USA) were used. CMMs are highly sophisticated mechatronic measuring devices used for highly accurate measurements. Most commonly, they have three axes (x, y, z), with a measuring probe attached to the end effector. The measuring probe collects data in the CMM coordinate system with high precision and can be controlled manually or by a computer.

The phantom target designs can further be divided into anatomical and non-anatomical, as previously suggested by Widmann et al. (2009). *Anatomical targets* usually present characteristic anatomical points on the skin and bone, or typical neurosurgical internal targets, e.g., tumor tissue (Liu et al., 2001; Arata et al., 2011; Niccolini et al., 2016). Anatomical targets are better at simulating a real scenario, but complicated to measure and more expensive to produce. In order to test a tumor removal procedure on anatomical targets, Arata et al. developed a plastic skull phantom filled with gelatin mimicking the brain tissue and a piece of pig brain tissue representing the tumor as an anthropomorphic target (Arata et al., 2011). A neurosurgical robot for brain tumor removal was tested on the phantom and error measurements were obtained using an optical measurement system (*Optotrak Certus, Northern Digital Inc., USA*). The phantom was used to simulate a complete brain tumor resection surgery, which is often a highly delicate and time-consuming procedure for neurosurgeons. The type of phantom designs using the actual brain tissue as target points and anthropomorphic skull replicas are suitable for simulating the anatomy and texture of the bone and the target point. The phantom drawbacks are the absence of an entry point and the inability to localize and measure the target point error unambiguously.

**Figure 6:**
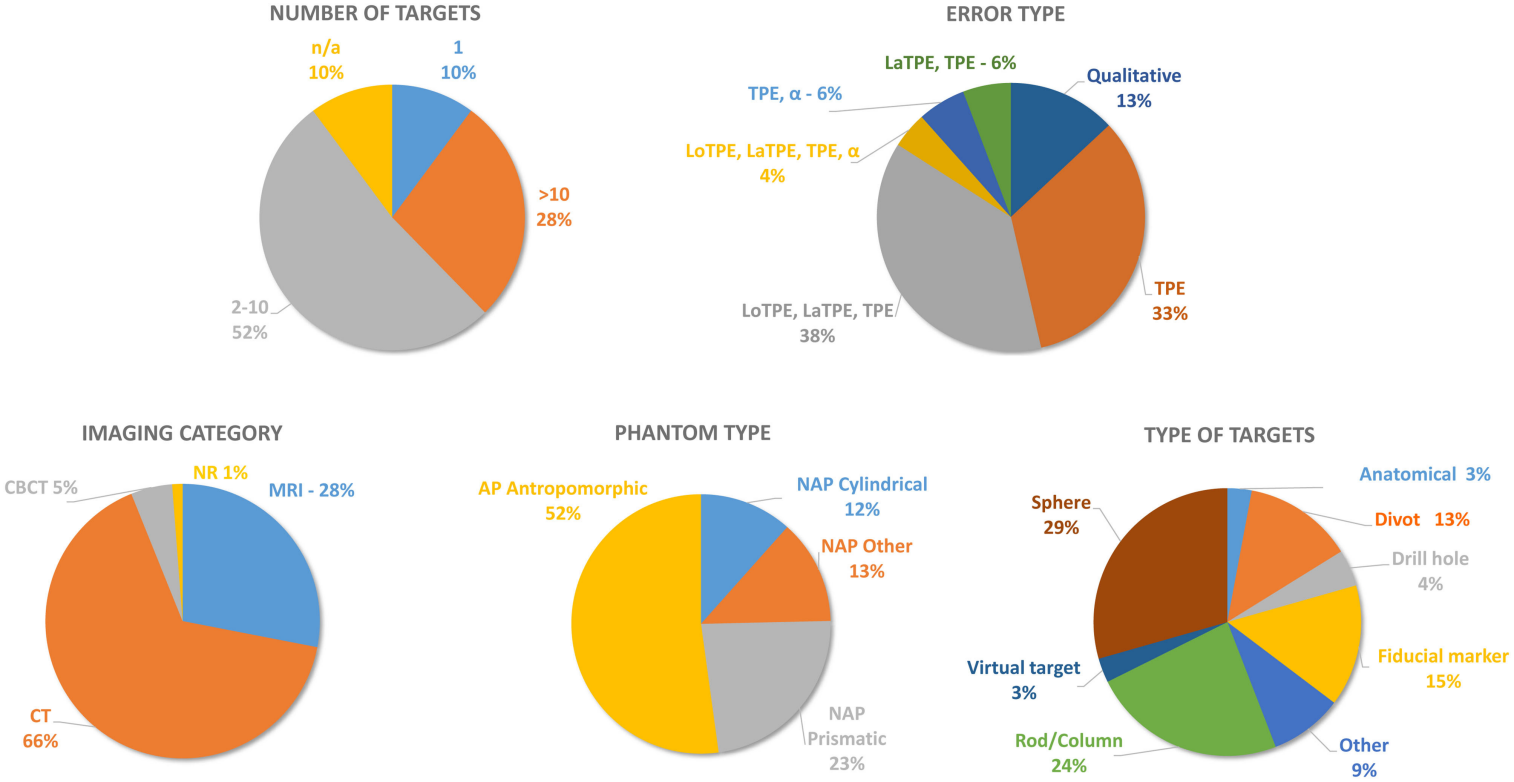
A statistical review of phantom designs according to the following criteria: number of targets, target point error type, imaging category, phantom type, and type of targets. EPE, entry point error; LaTPE, lateral target point error; LoTPE; longitudinal target point error; TPE, total target point error; α, angular error; CBCT, cone beam computerized tomography; CT, computerized tomography; MRI, magnetic resonance imaging; NR, not reported; NAP, non-anthropomorphic phantom.

*Non-anatomical targets* are more common since they can be economically produced and easily measured, but they lack the ability of simulating the target anatomy properly. Most common shapes of non-anatomical targets are spheres, rods, and divots (see Figure 6 and Table 1). Meng et al. developed an organic glass phantom with non-anatomical targets in order to measure the application accuracy of a surgical robot for SEEG (Meng et al., 2014) (Figure 5G). The phantom encompasses a volume similar to a human head volume. It was made as a hollow cylinder with target columns distributed across its base. Thirteen chamfers on vertical cylinders were used as target points. A Polaris optical tracking system (*Northern Digital Inc., Waterloo, Canada*) and a CMM were used to measure the robot targeting error. The main disadvantages of the phantom design are its cylindrical shape and the lack of EPE measurement. Additionally, the target points made as chamfers are inferior to spherical targets due to their inaccurate localization in CT scans. A different type of non-anthropomorphic targets was developed by Tovar-Arriaga et al. (Figure 5E): nine rods with tips of different heights distributed on a plastic phantom base in different positions simulated the targets (Tovar-Arriaga et al., 2011). That non-anthropomorphic phantom was used in measuring the targeting error of a robotic system based on a DLR/KUKA Light-weight robot III (*KUKA AG, Augsburg, Germany*). The robot targeting error was measured using a robot-driven angiographic C-arm system (*Artis Zeego, Siemens, Healthcare, Forchheim Germany*). The disadvantage of that phantom is in the “tip” shape of the target points; this tip shape is difficult to precisely plan and localize preoperatively on CT scans. Furthermore, the EPE and α measurements could not be taken on this phantom.

### Compatibility With Standard Stereotactic Frames and Head Holders

Stereotactic frames are manual aiming devices used in the stereotactic neuro-navigation operation workflow; nowadays, they are still considered as the gold standard for precise stereotactic neurosurgical targeting such as DBS surgery procedures (Chudy et al., 2018). To conduct stereotactic frame accuracy testing, the phantom should be rigidly fastened to a stereotactic frame by head pins in order to simulate the frame-based operation workflow. In order to achieve a satisfying rigid connection, three or four head pins are used, depending on the system. A non-deformable and rigid phantom material is essential. The most commonly used materials in phantom designs are Plexiglas and hard plastics due to their weight-to-mechanical rigidity ratio and imaging compatibility. Stereotactic frames ensure a very rigid and robust connection to the patient's head. Most commonly used frames are the Leksell, Cosman-Robert-Wells, and Zamorano-Dujovny frames (Table 1).

### Compatibility With Standard Localization and Registration Methods

Localization is the process of determining the spatial coordinates of specific features in the physical space using special devices, such as cameras, lasers, and tracking devices, or, in the image space, using CT or MRI. Registration is the process of calculating a geometric transformation that aligns a view of the object from the image space with the view of the same object in the physical space (Šuligoj et al., 2018a,b). Registration accuracy greatly depends on the type of methods and references used for localization. Registration methods classified according to the used marker type can be extrinsic, intrinsic, and non-image based (calibrated coordinate systems) (Markelj et al., 2012). Extrinsic methods use externally attached objects, such as bone-attached fiducial markers, skin-affixed fiducials, and different types of stereotactic frames. Intrinsic methods use the patient's anatomical landmarks on the outer surface of the head (and face) or inner brain structures for registration, while calibration-based methods use information acquired from imaging devices, pre-calibrated in reference to the operation table. In clinical practice, the three most widely used approaches in neuro-navigation system registration are frame-based, fiducial, and surface-matching registrations (Fitzpatrick, 2010). Frame-based registration uses a stereotactic frame rigidly fixed to the patient (or phantom) with different types of localizers for CT or MRI visibility. Fiducial-based registration uses different types of fiducials which are fixed to the patient's skin (adhesive fiducials) or to the bone (bone fiducials). The fiducials are localized in the preoperative image and also in the operating theater on the actual patient, and a registration is calculated. Surface matching is a type of markerless registration that uses the surface of the head and parts of the face. In markerless registration, an intraoperative image of the patient acquired by a neuro-navigation system or an intraoperative CT/MRI is registered to the preoperative CT or MRI scan.

To demonstrate the compatibility with standard registration and localization methods, a phantom should accurately simulate the registration characteristics of a real case/patient. If extrinsic methods are used, the phantom should use the same spatial distribution of fiducial markers, the number of fiducials, and the localization method as in real cases (Smith et al., 2014). For intrinsic methods, the surface of the phantom or the internal brain regions used for localizing anatomical points should have the shape and the area size similar to those in real cases (Lefranc et al., 2014). Registrations based on pre-calibrated imaging devices should use the same fixed positions of the operation table and the phantom with respect to the imaging device during image acquisition (Markelj et al., 2012). A phantom which is compatible with the majority of localization methods was developed by Lefranc et al. (2014) (Figure 5C). The phantom uses a removable and water fillable container for measuring the accuracy of the ROSA brain robotic system. The phantom was designed as a face replica, with a cavity (wall thickness of 0.25 mm) that could be filled with a contrast agent for surface markerless registration. That phantom is also compatible with both the CT and MRI preoperative imaging techniques. Twenty plastic tubes fillable with water or contrast agent were used as target points for measurements of the ROSA brain system conducted through a postoperative and intraoperative flat panel CT scans (*O-arm CT scanner, Medtronic, Minneapolis, Minn., USA*). Concerning its versatility, this phantom can be used for broad types of percutaneous operations, such as brain biopsies, DBS surgery, and ventricular drainage. An additional specialty of this phantom is its ability to be localized and registered with fiducial-based, frame-based, and frameless surface registration techniques representing all major types of localization modalities in image-guided neurosurgery. The main advantages of this phantom design are the anthropomorphic shape, the specially designed face for surface recognition, and the removable water compartment. Using tubes as target points is its main disadvantage due to the imprecise definition of the target center in the preoperative phase and the absence of entry points for the EPE and α measurement.

### Compatibility With Devices for Measuring the Targeting Error

The targeting error measurements can be obtained by using contact methods (mechanical and electromechanical measurement systems, and stereotactic frames) and non-contact ones (microscopes, frameless image-guided systems, vision systems, and imaging systems).

Contact measuring systems include Vernier calipers, metal rulers, CMM, and standard stereotactic frames (Table 1). In a great number of phantom designs, contact measurements are not possible (narrow measuring spaces, the phantom filled with gelatin, etc.), and in these cases, non-contact methods must be used.

In non-contact methods, measurements are obtained without a physical contact with the measured object by means of an optical device, a scanning device, or an imaging system. The main advantage of these methods is the lack of physical contact with the measured object; consequently, no additional measuring error is introduced through physical interference. Non-contact measuring systems include microscopes, frameless image-guided systems, such as Polaris, Stealth Station, Stryker Leibinger, and Varioguide, and imaging systems such as a CT or MRI (Table 1). The majority of non-contact systems have a certain intrinsic error in relation to the resolution of the image, the calibration of the system, the triangulation (when stereo systems are used), and the noise (considering that the ambient conditions are not perfect). Standard imaging methods such as CT and MRI have geometric errors of ~0.3 mm or greater (Lefranc et al., 2014), while microscopes can only measure deviations in a plane, i.e., only the lateral or the longitudinal error. Given all previously mentioned characteristics and types of errors, all non-contact measurement system have certain drawbacks and limitations.

Taking into account that high-precision contact measuring systems such as CMM have errors at a micron level, they hold a dominant position in the absolute accuracy domain. However, considering that most phantom trials are done in the operating theaters where CMM machines cannot be deployed or where it is not possible to come into contact with the tool, a compromise between practical aspects and the accuracy needs to be taken into account.

## Discussion

Prior to clinical utilization of robotic, frameless, and frame-based stereotactic neuro-navigation systems, studies have to be carried out on phantoms to ensure patient safety. The main purpose of these studies is to evaluate the targeting accuracy of neuro-navigation systems within the intracranial space and to measure their targeting errors (TPE, EPE, α). The value of a phantom investigated in a study is higher if the phantom provides a human-like anatomy and if a realistic simulation of the procedure can be performed. We have summarized essential and relevant features of phantom designs in more detail in the Supplementary Table 1. In Figure 4 we have summarized the four main categories of neuro-navigation systems for which phantoms were developed in the period from 2000 to 2020. Although the phantoms are being developed to test specific neuro-navigation systems, the same phantom can be used for a number of different neuro-navigation systems (robotic, stereotactic frame-based and frameless). Figure 4 also shows the trend in the development of stereotactic neuro-navigation, showing an intensified development of robotic neuro-navigation systems.

The working volume of most phantom designs is approximately the size of the human head. The anthropomorphic phantoms can accurately represent the anatomy, but it is very difficult to accurately simulate soft tissues (Landi et al., 2001; Liu et al., 2001; Li et al., 2002; Henderson, 2004; Henderson et al., 2004; Labadie et al., 2005; Pappas et al., 2005; Shamir et al., 2006; Eljamel, 2007; Rachinger et al., 2007; Widmann et al., 2008; Xia et al., 2008; Arata et al., 2011; Joskowicz et al., 2011; Comparetti et al., 2012; Larson et al., 2012; Lefranc et al., 2014; Kajita et al., 2015; Ballesteros-Zebadúa et al., 2016; Lin et al., 2016; Cardinale et al., 2017; Cutolo et al., 2017; Zeng et al., 2017). Although anthropomorphic phantoms can better simulate the operative procedure, non-anthropomorphic phantoms are also being developed and used to almost the same extent as anthropomorphic ones because they can be easily produced and are superior in target error measurement (Figure 6) (Moriarty et al., 2000; Benardete et al., 2001; Poggi et al., 2003; Krempien et al., 2004).

We have observed that the number of targets in phantom designs ranges from one to sixty-four, with the highest percentage (49%) of targets between two and ten (Figure 6). Regarding the target shape, a slight predominance of spherical targets can be noted; this is accounted for by the fact that it is possible to pinpoint the center of the circle in every cross-section of the imaged volume. The target material ranges from Plexiglas, to titanium or alumina oxide spheres, metal tips, animal brain, gelatin, ceramics, and plastics (Table 1). When designing a phantom, it is useful to include a larger number of target points so that several measurements with different trajectories can be conducted on the same phantom. A phantom should be filled with agent-enhanced water (Carter et al., 2000; Steinmeier et al., 2000; Benardete et al., 2001; Mutic et al., 2001; Yu et al., 2001a,b; Lavely et al., 2004; Wang et al., 2004; Isambert et al., 2008; DeWerd and Kissick, 2014; Lefranc et al., 2014; Nakazawa et al., 2014) or some other MRI compatible medium to improve imaging visibility (Moriarty et al., 2000; Widmann et al., 2009; Schouten et al., 2010; Larson et al., 2012; Squires et al., 2014; Li et al., 2015) and should be compatible with standard stereotactic frames and head holders in terms of the need to withstand great forces. The majority of stereotactic procedures, such as DBS and brain biopsies, are still performed using a stereotactic frame. The compatibility of phantoms with stereotactic frames is therefore important for stereotactic neuro-surgery. In order for a phantom to be compatible with different imaging methods such as the most commonly used CT and MRI, the phantom filling should closely replicate the brain tissue which has a high water percentage of ~73%. For CT imaging, water is used to properly simulate the attenuation of x-ray beams in order to get a realistic contrast between the implanted targets and the surrounding tissue. For MRI visibility, it is crucial to have the cranial cavity filled with water in order to get a proper scan. The correspondence between relevant features of a phantom and an actual patient in the clinical environment is required when connecting *in vitro* and *in vivo*. Filling, as one of design features is important because it has to represent the brain tissue in the best possible way. Several filling materials were identified in previously reported papers, such as water, agar gel, and contrast-based solutions; advantages of these fillings are easy preparation and imaging characteristics that enable close similarities with clinical scenarios.

Searching through the literature, we have noticed that the TPE measurements were performed either quantitatively as the total TPE or through its components, i.e., the lateral (LoTPE) and the longitudinal error (LaTPE) (Figure 6, Table 1). Concerning the EPE, we have identified a lack of quantitative measurement of entry point errors as one of the main issues of the current phantom designs (Table 1). In our opinion, the EPE is also one of the key errors in stereotactic neuro-navigation procedures, and TPE measurements are not sufficient for providing precise information about the overall error (see Figures 2B,C). In actual clinical procedures, the entry point error, due to which the surgical instrument deviates from the planned trajectory, can significantly influence the target point error. Drill tilt caused by shear forces in non-perpendicular trajectories on the cranial bone can lead to greater entry point errors. This is reported in literature (Cardinale et al., 2013; Dlaka et al., 2018) and for this reason we suggest that a stereotactic phantom should have the ability of quantitative measurement of the EPE. The quantitative measurement of the EPE enables better understanding of all the errors of a tested neuro-navigation device (robotic, frame-based, and frameless).

In the information obtained through the literature review, we have identified two main research issues. First, there is no adequate phantom design that can be used for simultaneous qualitative measurements of TPE and EPE. The phantom design should be well suited for almost all of the currently available localization methods, imaging techniques, and registration methods. We published a paper on a conceptual design of a phantom (called the T-phantom) that can be used for measuring the TPE and EPE (Švaco et al., 2016). Our initial phantom concept was suitable for CT imaging but had certain limitations regarding the localization of entry and target points. The T-phantom is a non-anthropomorphic phantom which is not suitable for markerless localization and registration. It cannot be filled with a water-based solution; consequently, an MRI scan of the phantom cannot be acquired. For adequate measurement of the target and entry point errors, development of a special stereovision non-contact measurement device is needed. A phantom that is anthropomorphic, that enables markerless registration, and that is suitable for MR imaging could be developed as an upgraded version of the initial T-phantom concept.

The second research issue identified through the literature review concerns the lack of a simple, portable, and accurate measuring device for error assessment. A number of papers report the use of stereotactic frames for error measurement (Landi et al., 2001; Willems et al., 2001; Yu et al., 2001b; Li et al., 2002; Rosenow and Sootsman, 2006; Eljamel, 2007; Heinig et al., 2011, 2012; Lefranc et al., 2014; Nakazawa et al., 2014; Kajita et al., 2015), but the intrinsic error of a stereotactic frame is considerable. For example, the widely used Leksell arc centered stereotactic frame has a 0.7 mm intrinsic error (Lozano, 2009). A number of researchers have used an intra or postoperative CT or MRI for error measurements, but imaging methods also have intrinsic geometric errors and are not capable of measuring accuracy below 0.3 mm (Lefranc et al., 2014). This type of error measurements introduces localization and registration errors, thus affecting the overall measurement reliability. The application of CMMs is limited in the operating theater while their price is significant. Furthermore, CMMs are mostly inflexible and robust and are unable to measure any arbitrary trajectory. We believe that a flexible and highly accurate optical system using standard industrial cameras could be developed for the qualitative measurement of EPE and TPE.

## Conclusions

This paper provides a systematic review of stereotactic neuro-navigation phantom designs and their relevant features. The paper also gives an overview of the targeting error measurement methodologies currently used in stereotactic neuro-navigation procedures. The review of phantom designs revealed that the majority of the designs have a suitable methodology for TPE measurement but lack objective measurements of EPE and α. Furthermore, there is a wide range of different localization devices and registration techniques incorporated in state-of-the-art stereotactic neuro-navigation systems.

A universal phantom design recommended by the society of computer-assisted surgery is needed; it should be compatible with most common imaging techniques (CT, MRI) and suitable for simultaneous qualitative measurement of TPE, EPE, and α.

## Data Availability Statement

All datasets generated for this study are included in the article/Supplementary Material.

## Author Contributions

MŠ designed the study and wrote the first version of the manuscript. MŠ, IS, DD, and FŠ conducted literature research, contributed to data analysis, and designed the figures and tables. BJ and DC interpreted the results and revised the manuscript. MR contributed to the study concept and design and revised the manuscript. All authors read and approved the final version of the manuscript as submitted.

## Conflict of Interest

The authors declare that the research was conducted in the absence of any commercial or financial relationships that could be construed as a potential conflict of interest.
