# Supplementary material for: Stereotactic Neuro-Navigation Phantom Designs: A Systematic Review
Source: Front Neurorobot. 2020 Oct 23;14:549603. doi: 10.3389/fnbot.2020.549603 (PMC7644893; doi:10.3389/fnbot.2020.549603)
Supplement: Supplementary file 1 [file Table_1.DOCX]

Supplementary Material

# Supplementary Table

Supplementary Table 1. Detailed literature review of phantom designs (from 2010 to 2020).

*Abbreviations: AP – Anthropomorphic Phantom; NAP – Non Anthropomorphic Phantom; NR – Not Reported; LLA – Lateral; Longitudinal and Absolute; LLAA – Lateral; Longitudinal, Absolute, and Angular; CMM – Coordinate Measuring Machine; FIGS – Frameless Image Guided System, ST – slice thickness, NUT – number of targets*

| Study | Size and shape | Material | Filling | Target design (NUT) | Error Measurement | Image data for navigation (ST) | Accuracy verification device | Phantom features |
| --- | --- | --- | --- | --- | --- | --- | --- | --- |
| Baron et al. (2010) | Accurately fabricated aluminium block, 125 × 75 × 35 mm (NAP) | Aluminum | Air | Center of plaster-filled holes 10mm in diameter (24) | Target: - Quantitative - LoTPE, LaTPE, TPE Entry:  - Quantitative | CT | - CMM (Zeiss ZMC 530, Carl Zeiss IMT GmbH, Oberkochen, Germany) | - Five threaded holes were added to mount 5 mm titanium spheres for registering the block-to-physical space - The exact positions of the spheres relative to the block were determined using a caliper (resolution 0.01 mm) |
| Baron et al. (2010) | Size not reported (NAP) | Acrylic | Air | Drill hole (1) | Target: - Quantitative - LaTPE, TPE Entry:  - Quantitative | CT | - Imaging system (Post-operative xCAT ENT CT, Xoran Technologies, Ann Arbor, MI, USA) | - Acrylic block with three embedded spherical fiducial markers  - Three spheres enable registration between the image and the physical space, immobilized by embedding in epoxy |
| Giese et al. (2010) | Size not reported (NAP) | Acrylic glass | Agarose (polysaccharide) | Plastic fiducial markers (11) | Target: - Quantitative - LoTPE, LaTPE, TPE, α Entry:  - Quantitative | 1.5T MR (2 mm) CT (0.625 mm) | - Imaging system (iPlan Cranial 2.6 (BrainLab)) | - Wedge-shaped phantom without agarose filling - The walls are made of transparent acrylic material, with 9 holes drilled for probe placement in the broader convex side. - For registration, 11 fiducial markers are glued directly to the model and the plate |
| Schouten et al. (2010) | Size not reported (NAP) | Plastic | Agar gel | Plastic spheres 2mm in diameter (NR) | Target: - Quantitative - LoTPE, LaTPE, TPE | MR T1 (NR) | - Imaging system (Postoperative MR imaging, Magnetom Trio, Siemens Medical Solutions, Erlangen, Germany) | - All spheres were embedded in the agar at the same depth of 3 cm, with a distance of 2–3 cm between them |
| Arata et al. (2011) | Anthropomorphic phantom | Plastic | Air | Pig brain tissue and gelatin (1) | NR: - Qualitative | MR (NR) | - Optical system (Optotrak Certus, NDI - Northern Digital Inc., USA) | - The phantom was developed using 3D optical lithography - The tumor part was filled with brain tissue from a pig to perform the tumor-removal procedure |
| Brodie and Eljamel (2011) | Anthropomorphic phantom | Synthetic | Air | Fiducial marker with a central empty hole (NR) | Target: - Quantitative - TPE Entry:  - Quantitative | CT (NR) | - Mechanical system (Submillimetric caliper) | - Synthetic human skull replica (SHSR) phantom  - Fiducials attached to the inner surface of the inner table of the SHSR were used as burr hole targets  - The fiducial on the surface was used as an entry point, and the burr hole targets were used as target points for the burr hole trajectory  - fiducials filled with radio-opaque material for CT visibility mixed with oil for MRI visibility |
| Joskowicz et al. (2011) | Anthropomorphic phantom | Plastic | Air | Metal disc, adhesive markers (2) | Target: - Quantitative - LaTPE, TPE | CT (NR) MR (NR) | - Imaging system (Postoperative CT and MR imaging) - Visual reading (Visual reading) | - Star-shaped mounting base  - Custom-made replica of a skull phantom model  - Two targets embedded in deep-brain locations - The center of each target is marked and 0.5 mm concentric circles are drawn, which allows estimating the deviation of the surgical tool from the planned trajectory - Targets are defined on the surface of the cortex with adhesive markers |
| Kratchman et al. (2011) | Overall dimensions: 150 mm × 150 mm × 38 mm (NAP) | Acrylic | Air | Fiducial marker spheres 3 mm in diameter (19) | Target: - Quantitative - TPE | CT (NR) | - Imaging system (CT scan, Xoran Techn., Ann Arbor, MI) - Mechanical system (FARO GagePlus, FARO Technologies Inc., Lake Mary, FL) | - The phantom is milled from an acrylic block |
| Tovar-Arriaga et al. (2011) | Size not reported (NAP) | Plastic | Air | Plastic rods with tips (9) | Target: - Quantitative - TPE | CT (NR) | - Imaging system (Artis zeego, Siemens Healthcare, Forchheim Germany) | - The phantom consists of nine rods with tips distributed in different positions - The height of the higher five rods was 40 mm - The height of the smaller four rods was 25 mm - The construction accuracy of the testing device is about 0.01 mm. |
| Comparetti et al. (2012) | Anthropomorphic phantom | Plastic | Polyvinyl alcohol | Gadolinium markers (2) | Target: - Quantitative - TPE, α Entry:  - Quantitative | T1 MR (1 mm) | - Optical system (Optotrak Certus, NDI) | - A plastic skull with polyvinyl alcohol as brain mimicking material - Two gadolinium markers (targets) are fixed on the base of the skull |
| Heining et al. (2012) | Anthropomorphic phantom | Plastic | Air | Plastic rods (6) | Target: - Quantitative - LoTPE, LaTPE, TPE | CT (0.4 mm) | - Optical system (Two perpendicularly mounted cameras: DFK 31AF03 TheImaging Source, Germany) | - Commercially available head phantom was equipped with six sharp-tipped plastic rods, representing the targets |
| Kronreif et al. (2012) | Size not reported (NAP) | Acrylic | Air | PMMA cylinder (rod) (9) | Target: - Quantitative - TPE Entry:  - Qualitative | CT (NR) MR (NR) | - Mechanical system (Digital calipers) - FIGS (VectorVision, BrainLab) | - A phantom that simulates different targeting options for brain biopsies - The top plate of the phantom has a set of openings, simulating possible entry positions |
| Larson et al. (2012) | Anthropomorphic phantom | Plastic | Gelatine and water | Ceramic disc (8) | Target: - Quantitative - TPE | 1.5T MR (NR) | - Imaging system (Intraoperative MR imaging: Philips Achieva, Best, The Netherlands) | - 2 SMART Frames, mounted on the phantom in a bilateral fashion - Ceramic discs (targets) are inserted into the phantom to a depth of < 90 mm |
| Gerber et al. (2013) | Approximate representation of the human mastoid 90 × 90 × 35 mm (NAP) | Acrylic | Air | Metal fiducial screws (10) | Target: - Quantitative - TPE | CBCT (0.15 mm) | - CMM (Tesa 3D Micro-MS454, Renens, Switzerland) | -10 fiducial screws are embedded in the phantom at different heights at varying insertion angles - Four 3.17 mm diameter aluminum spheres used as landmarks |
| Schulz et al. (2013) | Sponge phantom with dimensions of 280 × 160 × 180 mm (NAP) | Sponge | Sponge | Plastic spheres (10) | NR:- Qualitative | CBCT (0.9 mm) | - Imaging system (C-arm CBCT device: Axiom Artis Zeego, Siemens Healthcare, Forchheim, Germany) | - 10 crosswise plastic targets (span length 1.7 cm, thickness 2 mm, center diameter 2 mm) |
| Lefranc et al. (2014) | Anthropomorphic phantom | Plastic | Water/contrast agent | Water fillable plastic tubes (20) | Target: - Quantitative - TPE | 3T MR T1 (1 mm) 3T MRT2 (1 mm) CT (0.625 mm) fpCT (0.8 mm) | - Imaging system (Postoperative CT imaging) - Imaging system (intraoperative fpCT imaging: Medtronic, Minneapolis, Minn., USA) | - A removable container can be filled with water - The face of the phantom is a cavity (wall thickness of 0.25 mm) that can be filled with contrast agent - In a CT scan, the phantom is easily viewed because of its radiopacity -The phantom was designed so that it could be used with all types of registration and preoperative imaging techniques |
| Meng et al. (2014) | Similar size to the human head (NAP) | Organic glass | Air | Pits on the top of slender columns, organic glass (13) | Target: - Quantitative - TPE Entry:  - Qualitative | CT (0.7 mm) | - Optical system (Polaris, NDI: Northern Digital Inc., Waterloo, Canada) - Optical system (Optotrak, NDI) | - Organic glass phantom  - A set of locating pits are distributed on the phantom |
| Gang Li et al. (2015) | Size not reported (NAP) | Gelatin | Gelatin | Plastic pins and arches (NR) | Target: - Quantitative - TPE, α | MR T2 (MR) | - Imaging system (Postoperative MR imaging) | - Standard phantom - Complex geometric features, including cylindrical cross section, arch and pin section |
| Kajita et al. (2015) | Anthropomorphic phantom | Plastic | Air | Metal spheres (6) | Target: - Quantitative - LoTPE, LaTPE, TPE Entry:  - Qualitative | CT (0.5 mm) | - Mechanical system (Digital caliper) | - Phantom is fitted with implantable markers - The markers are used to accurately measure the location of probe tip placed according to each of the frame-based methods |
| Šuligoj et al. (2015) | A calibration board phantom 250 × 250 mm (NAP) | Plastic | Air | Holes in plastic (64) | Target: - Quantitative - LoTPE, LaTPE, TPE | CT (0.75 mm) | - Optical system (Infrared tracking system: Polaris Vicra, NDI) - Imaging system (Siemens Sensation 16 CT scanner) | - The phantom has 8 × 8 holes positioned at 30 mm distances in perpendicular directions - Calibration board holes are designed to precisely center the tapered tip of the marker probe |
| Ballesteros-Zebadúa et al. (2016) | Anthropomorphic phantom | Tissue and bone radio-equivalent polymers | Air | Acrylic rods and pyramids (6) | Target: - Quantitative - TPE | CT (0.7 mm) | - Optical system (Calibrated pointer and a camera tracking system) | - The phantom is designed for radiation dosimetry made of tissue and bone radio transparent polymers(Radiosurgery Head Phantom, USA) - Custom-built accessory kit consisting of different acrylic shapes - Six targets with different shapes were included in different locations inside the phantom cranial cavity |
| Lin et al. (2016) | Anthropomorphic phantom | Plastic | Air | Plastic drill holes (NR) | Target: - Quantitative - LoTPE, LaTPE, TPE | CT | - Mechanical system (3D haptic device, PHANTOM Omni model; SensAble Technologies Inc., Wilmington, MA, US) | - A 3D–printed phantom (patient's head replica) with medical markers |
| Niccolini et al. (2016) | Size not reported (NAP) | Paper | Air | Ink-printed point on paper (9) | Target:- Quantitative- LaTPE, TPE | CT (NR) | - Microscope (KH7700 , Hirox Co., Tokyo, Japan) | - A paper sheet with nine printed target points- The target plane can be inclined in order to obtain multiple phantom poses |
| Niccolini et al. (2016) | Anthropomorphic phantom | Plastic | Air | Plastic 3d printed model of cerebral ventricular system (1) | Target:  - Qualitative - Qualitative Entry:  - Qualitative | CT (NR) | - Imaging system (Intraoperative imaging using an endoscope camera) | - A standard phantom (3B Scientific) was modified by inserting a passing through channel for the endoscope  - A printed 3D ventricular model was fixed to a phantom |
| Švaco et al. (2016) | Size not reported (NAP) | Plexiglass | Air | SLS plastic printed hollow cylinder (8) | Target: - Quantitative - LoTPE, LaTPE, TPE, α Entry:  - Quantitative | CT (NR) | - Optical system (Custom-built stereovision system with two peprendicular industrial cameras) | - Four trajectories perpendicular to the base plate and four tilted trajectories - The center of each hollow cylinder pair is used as a target and an entry point - Longitudinal and lateral errors are measured by a stereovision system - Angular error is calculated from the target and entry point lateral errors |
| Cardinale et al. (2017) | Anthropomorphic phantom | Plastic | Air | Plastic/Ruby sphere (10) | Target: - Quantitative - LoTPE, LaTPE, TPE Entry:  - Quantitative | CBCT (NR) | - Imaging system (Postoperative CBCT) | - Humanlike plastic skull without the cranial vault - Ten internal fiducial markers (Cranial Marker System, Leibinger) were fixed to the skull - Each fiducial marker is made of a cranial screw supporting a removable CT-visible target |
| Cifuentes et. al. (2017) | Cylinder shape phantom (height 130 mm, diameter 140 mm) (NAP) | Acrylic | Air, water | Acrylic rods (10) | Target: - Quantitative - TPE | CT (1 mm) | - Optical system (Two optical sensors of the NeuroCPS system) | - The superior cover can be removed in order to reach the internal targets - Ten adhesive fiducial markers are mounted on the lateral surface for the registration procedure |
| Cutolo et. al. (2017) | Anthropomorphic phantom | 3d printed plastic/ceramic clay, liquid polymer/skin-like silicon layer | PVA-C-based hydrogel | 3D printed Plastic sphere (4) | Target:  - Qualitative Entry:  - Qualitative | CT (1.25 mm) | Visual reading | -The skull base is embedded with bilateral frontal lesions both medial to the adjacent eloquent areas (Eloq. area) -The inner surface of the skull base represents several housing designed to insert further lesions or eloquent areas - liquid polymer is used for brain representation - silicone rubber is used to simulate the skin |
| Rau et .al. (2017) | Size not reported (NAP) | Vinyl sheet | Air | Vinyl divots (18) | Target: - Quantitative - LaTPE, TPE | CT (NR) | - Microscope (Leica APO Z6, Leica Microsystems GmbH, Wetzlar, Germany) | - A target plate (vinyl sheet) with a grid of CNC milled reference marks with an intermediate distance of 10 mm - A metal spike is inserted through the bushing marking the reached target point with an indentation in a vinyl sheet at the base plate of the test bench |
| Šuligoj et al. (2017) | Size not reported (NAP) | Plastic | Air | Plastic retroreflective spheres (10) | Target: - Quantitative - TPE | CT (0.7 mm) | - Optical system (Infrared tracking system: Polaris, NDI) | - The phantom has a polymer base with one central pillar and 10 spherical fiducials - The center of each spherical fiducial is used as a target point |
| Zeng et al. (2017) | Anthropomorphic phantom | 3d printed plastic | Air | Plastic rod (13) | Target: - Quantitative - TPE Entry:  - Quantitative | CT (0.8 mm) | - Optical system (projector-camera system) | - The phantom originally generated from CT data of a patient  -13 targets on a plastic plate with different heights |
| Guo et al. (2018) | Anthropomorphic phantom | Plastic | Agar-agar gel | Virtual targets - Agar agar (10) | Target: - Quantitative - LoTPE, LaTPE, TPE | MR T2 (NR) | - FIGS (EM positional tracking system (Aurora, NDI Medical,  Canada)) | - Ten points were simulated as the STN targets, five at each side of a plastic plate.  - Measured targets coordinates were registered with the robot coordinate system. |
| Meng et al. (2018) | Anthropomorphic phantom | Plastic 3D printing | Air | Conical pivot on the top of each cylinder, plastic 3D printing (13) | Target: - Quantitative - TPE | CT (0.8 mm) | - Optical system (Multi-view stereo vision system containing two CCD cameras (CM3-U3-28S4CCS, Point Grey Research, Canada)) | - Phantom for automatic markerless registration testing - Head phantom consisting of a scalp surface shell and 13 internal targets on a base - The targets were randomly distributed inside the head  - Assembly and disassembly repeatability of 0.21mm. |
| Krüger et al. (2018) | Anthropomorphic phantom | 3D printed plastered skull | 10% agar | Lead targets in agar-agar (2) | Target:  - Qualitative Entry:  - Qualitative | CT (NR) MR T1 (NR) | - Imaging system (64-slice CT scanner  (Definition AS, Siemens) 3T MRI (Magnetom Prisma, Siemens), 3D digital X-ray system (Allura Xper FD20, Philips Healthcare)) | - The phantom can be used for the training of SEEG procedures |
| Kaushik et al. (2018) | Size not reported (NAP) | Spherical jar phantom with concentric shells | Air | Plastic hollow plastic pairs of cylinders   (NR) | Target:  - Qualitative Entry:  - Qualitative | CT (NR) | - Visual reading (Visual inspection) | - A glass jar phantom has two shells: the outer shell represents the skull and the inner shell represents the deeper zone (50 mm inside the brain) - Numerous pairs of collinear holes are made on both the outer and the inner shells which are in line with the center of the outer glass jar |
| Batista et al. (2019) | Anthropomorphic phantom | 3D printed plastic | Agar-agar and Copper and barium Sulfate solutions | Cylinders (14) and 3D printed plastic truncated spheres (10) | Target: - Quantitative - TPE, α | CT (1 mm) MR T1 (1 mm) | - FIGS (Polaris optical tracking system, NDI) | - The base of the phantom was based on the Leksell frame to allow the solid attachment of a computed tomography (CT) localizer.  - Each sphere has a different hyperbolic trajectory to its center, to simulate possible surgical challenges |
| Kaushik et al. (2019) | Anthropomorphic phantom | Plastic | Air | Plastic markers and holes of different sizes (NR) | Target:- Quantitative- LoTPE, LaTPE, TPE | CT (0.625 mm) | - Optical system (Digital camera (Dino-Lite Edge AM7115MZTL)) | - Standard skull phantom with drilled holes and fixed fiducial markers - Fiducial markers are used as target points and for registration |
| Kaushik et al. (2020) | Size not reported (NAP) | Biological (carrot) | Biological (carrot) | Spherical Mint (Pudin-Hara tablet) capsule 1.5mm in diameter (3) | Target: - Qualitative | micro-CT (0.15) | Visual reading | - A carrot was subjected to robotic neuronavigation testing - Mint (Pudin- Hara tablet) spherical capsules placed at three different locations within the carrot |

**REFERENCES**

Batista, P., Machado, I., Roios, P., Lavrador, J., Cattoni, M., Martins, J., et al. (2019). Position and orientation errors in a neuronavigation procedure: a stepwise protocol using a cranial phantom. *World Neurosurg*. 126, e342–e350. doi: 10.1016/j.wneu.2019.02.052

Faria, C., Erlhagen, W., Rito, M., De Momi, E., Ferrigno, G., and Bicho, E. (2015). Review of robotic technology for stereotactic neurosurgery. *IEEE Rev. Biomed.* Eng. 8, 125–137. doi: 10.1109/RBME.2015.2428305

Grimm, F., Naros, G., Gutenberg, A., Keric, N., Giese, A., and Gharabaghi, A. (2015). Blurring the boundaries between frame-based and frameless stereotaxy: feasibility study for brain biopsies performed with the use of a head-mounted robot. *J. Neurosurg*. 123, 737–742. doi: 10.3171/2014.12.JNS141781

Grunert, P., Darabi, K., Espinosa, J., and Filippi, R. (2003). Computeraided navigation in neurosurgery. *Neurosurg. Rev*. 26, 73–99. doi: 10.1007/s10143-003-0262-0

Guo, Z., Ziyang, D., Kit-Hang, L., Chim, L. C., Hing-Choi, F., Justin, D. L., et al. (2018). Compact design of a hydraulic driving robot for intraoperative MRI-guided bilateral stereotactic neurosurgery. *IEEE Robot. Autom. Lett*. 3, 2515–22. doi: 10.1109/LRA.2018.2814637

Hall, W. A., Galicich, W., Bergman, T., and Truwit, C. L. (2006). 3-Tesla intraoperative MR imaging for neurosurgery. *J. Neurooncol*. 77, 297–303. doi: 10.1007/s11060-005-9046-4

Hall, W. A., Liu, H., Martin, A. J., Maxwell, R. E., and Truwit, C. L. (2001). Brain biopsy sampling by using prospective stereotaxis and a trajectory guide. *J. Neurosurg.* 94, 67–71. doi: 10.3171/jns.2001.94.1.0067

Holloway, K. L., Gaede, S. E., Starr, P. A., Rosenow, J. M., Ramakrishnan, V., and Henderson, J. M. (2005). Frameless stereotaxy using bone fiducial markers for deep brain stimulation. *J. Neurosurg*. 103, 404–413. doi: 10.3171/jns.2005.103.3.0404

Jerbić, B., Nikolić, G., Chudy, D., Švaco, M., and Šekoranja, B. (2015). Robotic application in neurosurgery using intelligent visual and haptic interaction*. Int. J. Simul. Model*. 14, 71–84. doi: 10.2507/IJSIMM14(1)7.290

Kaushik, A., Dwarakanath, T. A., and Bhutani, G. (2018). Autonomous neuro-registration for robot-based neurosurgery. Int. J. Comput. Assist. Radiol.Surg. 13, 1807–1817. doi: 10.1007/s11548-018-1826-3

Kaushik, A., Dwarakanath, T. A., Bhutani, G., Moiyadi, A., and Chaudhari, P. (2020a). Validation of high precision robot-assisted methods for intracranial applications: preliminary study. *World Neurosurg*. 137, 71–77. doi: 10.1016/j.wneu.2020.01.206

Kaushik, A., Dwarakanath, T. A., Bhutani, G., and Srinivas, D. (2020b). Robot-based autonomous neuroregistration and neuronavigation: implementation and case studies. *World Neurosurg*. 134, e256–71. doi: 10.1016/j.wneu.2019.10.041

Krüger, M. T., Coenen, V. A., Egger, K., Shah, M., and Reinacher, P. C. (2018). Development of a standardized cranial phantom for training and optimization of functional stereotactic operations*. Stereotact Funct Neurosurg*. 96, 190–96. doi: 10.1159/000489581

Lefranc,M., Capel, C., Pruvot-Occean, A. S., Fichten, A., Desenclos, C., Toussaint, P., et al. (2015). Frameless robotic stereotactic biopsies: a consecutive series of 100 cases. *J. Neurosurg*. 122, 342–352. doi: 10.3171/2014.9.JNS14107

Lefranc, M., and Le Gars, D. (2012). Robotic implantation of deep brain mulation leads, assisted by intra-operative, flat-panel CT*. Acta Neurochir.* 154, 2069–2074. doi: 10.1007/s00701-012-1445-7

Marcus, H. J., Vakharia, V. N., Ourselin, S., Duncan, J., Tisdall, M., and Aquilina, K. (2018). Robot-assisted stereotactic brain biopsy: systematic review and bibliometric analysis. *Childs Nerv. Syst*. 34, 1299–1309. doi: 10.1007/s00381-018-3821-y

Mascott, C. R. (2005). Comparison of magnetic tracking and optical tracking by simultaneous use of two independent frameless stereotactic systems. *Operat. Neurosurg*. 57, 295–301. doi: 10.1227/01.NEU.0000176411.55324.1E

Meng, F., Fangwen, Z., Bowei, Z., Hui, D., and Wang, G. (2018). An automatic markerless registration method for neurosurgical robotics based on an optical camera*. Int. J. Comput. Assist. Radiol. Surg*. 13, 253–65. doi: 10.1007/s11548-017-1675-5

Minchev, G., Kronreif, G., Martínez-Moreno, M., Dorfer, C., Micko, A., Mert, A., et al. (2016). A novel miniature robotic guidance device for stereotactic neurosurgical interventions: preliminary experience with the iSYS1 robot. *J. Neurosurg.* 126, 985–996. doi: 10.3171/2016.1.JNS152005

Motkoski, J.W., and Sutherland, G. R. (2016). “Why robots entered neurosurgery,” in *Experimental Neurosurgery in Animal Models*, ed. M. Janowski (Springer: New York), 85–105. doi: 10.1007/978-1-4939-3730-1_6

Paleologos, T. S., Dorward, N. L., Wadley, J. P., and Thomas, D. G. (2001). Clinical validation of true frameless stereotactic biopsy: analysis of the first 125 consecutive cases. *Neurosurgery* 49, 830–835. doi: 10.1227/00006123-200110000-00009

Smith, J. A., Jivraj, J., Wong, R., and Yang, V. (2016). 30 Years of neurosurgical robots: review and trends for manipulators and associated navigational systems. *Ann. Biomed. Eng*. 44, 836−46. doi: 10.1007/s10439-015-1475-4

Spyrantis, A., Cattani, A., Strzelczyk, A., Rosenow, F., Seifert, V., and Freiman, T.M. (2018). Robot-guided stereoelectroencephalography without a computed tomography scan for referencing: analysis of accuracy. *Int. J. Med. Robot.* 14, 1–6. doi: 10.1002/rcs.1888

Švaco, M., Šekoranja, B., Šuligoj, F., and Jerbić, B. (2014). “Calibration of an industrial robot using a stereo vision system,” in *Procedia Engineering*, 459–463. doi: 10.1016/j.proeng.2014.03.012

Tan, A., Ashrafian, H., Scott, A. J., Mason, S. E., Harling, L., Athanasiou, T., et al. (2016). Robotic surgery: disruptive innovation or unfulfilled promise? A systematic review and meta-analysis of the first 30 years. *Surg. Endosc*. 30, 4330–52. doi: 10.1007/s00464-016-4752-x

Terrier, L., Gilard, V., Marguet, F., Fontanillers, M., and Derrey, S. (2019). Stereotactic brain biopsy: evaluation of robot-assisted procedure in 60 patients. *Acta Neurochir*. 161, 545–552. doi: 10.1007/s00701-019-03808-5

Wang, M. N., and Song, Z. J. (2011). Classification and analysis of the errors in neuronavigation. *Neurosurgery* 68, 1131–1143. doi: 10.1227/NEU.0b013e318209cc45

Widmann, G., Widmann, R., Widmann, E., Jaschke, W., and Bale, R. (2007). Use of a surgical navigation system for CT-guided template production*. Int. J. Oral Maxillofac. Implants*. 22, 72–8.

Widmann, G., Widmann, R., Widmann, E., Jaschke, W., and Bale, R. J. (2005). In vitro accuracy of a registration technique for imageguided template production*. Clin. Oral Implants. Res*. 16, 502–8. doi: 10.1111/j.1600-0501.2005.01131.x

Wolfsberger, S., Rössler, K., Regatschnig, R., Ungersböck, K. (2002). Anatomical landmarks for image registration in frameless stereotactic neuronavigation. *Neurosurg. Rev*. 25, 68–72. doi: 10.1007/s10143-001-0201-x

Yasin, H., Hoff, H. J., Blümcke, I., and Simon, M. (2019). Experience with 102 frameless stereotactic biopsies using the neuromate robotic device. *World Neurosurg*. 123, e450–e456. doi: 10.1016/j.wneu.2018. 11.187
